# Human Metapneumovirus Infection in Wild Mountain Gorillas, Rwanda

**DOI:** 10.3201/eid1704.100883

**Published:** 2011-04

**Authors:** Gustavo Palacios, Linda J. Lowenstine, Michael R. Cranfield, Kirsten V.K. Gilardi, Lucy Spelman, Magda Lukasik-Braum, Jean-Felix Kinani, Antoine Mudakikwa, Elisabeth Nyirakaragire, Ana Valeria Bussetti, Nazir Savji, Stephen Hutchison, Michael Egholm, W. Ian Lipkin

**Affiliations:** Author affiliations: Columbia University, New York, New York, USA (G. Palacios, A. Valeria Bussetti, N. Savji, W.I. Lipkin);; University of California, Davis, California, USA (L.J. Lowenstine, M.R. Cranfield, K.V.K. Gilardi);; Mountain Gorilla Veterinary Project, Davis (L. Spelman, M. Lukasik-Braum, J.-F. Kinani);; Rwanda Development Board, Kigali, Rwanda (A. Mudakikwa, E. Nyirakaragire);; 454 Life Sciences, Branford, Connecticut, USA (S. Hutchison, M. Egholm)

**Keywords:** Viral disease, respiratory tract diseases, pneumonia, human metapneumovirus, gorilla, Gorilla beringei beringei, viruses, Rwanda, dispatch

## Abstract

The genetic relatedness of mountain gorillas and humans has led to concerns about interspecies transmission of infectious agents. Human-to-gorilla transmission may explain human metapneumovirus in 2 wild mountain gorillas that died during a respiratory disease outbreak in Rwanda in 2009. Surveillance is needed to ensure survival of these critically endangered animals.

The world’s remaining 786 mountain gorillas (*Gorilla beringei beringei*) live in 2 parks in Rwanda, Uganda, and the Democratic Republic of the Congo. An ecotourism industry for viewing human-habituated mountain gorillas in the wild is thriving in all 3 countries. Mountain gorilla tourism helps ensure the sustainability of the species by generating much-needed revenue and increasing global awareness of the precarious status of this species in the wild. Tourism, however, also poses a risk for disease transmission from humans to the gorillas.

Habitat encroachment and poaching are threats to wildlife survival, particularly in the developing world. Mountain gorillas face an additional threat from infectious diseases. Second only to trauma, infectious diseases, primarily respiratory, account for 20% of sudden deaths (*1*). The close genetic relatedness of mountain gorillas and humans has led to concerns about the potential interspecies transmission of infectious agents (*2**,**3*). Although most surveillance efforts focus on risk for humans, mountain gorillas are immunologically naive and susceptible to infection with human pathogens. The parks in which mountain gorillas live are surrounded by the densest human populations in continental Africa. In addition, research and gorilla ecotourism brings thousands of persons from the local communities and from around the world into direct and indirect contact with the gorillas. The frequency and closeness of contact is particularly pronounced in Virunga National Park, where 75% of mountain gorillas are habituated to the presence of humans.

To minimize the threat of disease transmission, the Rwandan, Ugandan, and Congolese governments restrict tourist numbers and proximity, and the Congolese wildlife authority mandates that masks be worn by persons visiting gorillas. Nonetheless, the frequency and severity of respiratory disease outbreaks among mountain gorillas in the Virunga Massif have recently increased. From May through August 2008, sequential respiratory outbreaks occurred in 4 groups of mountain gorillas accustomed to tourism in Rwanda. Between June 28 and August 6, 2009, a fifth outbreak occurred in 1 of these groups, Hirwa. We describe the Hirwa outbreak. Respiratory outbreaks were defined as more than one third of animals in a group exhibiting signs of respiratory disease (coughing, oculonasal discharge, and/or lethargy).

## The Cases

The Hirwa group consisted of 12 animals: 1 adult male, 6 adult females, 3 juveniles, and 2 infants. Moderate to severe respiratory disease (>2 characteristic signs) developed in 11 of 12 animals. Five (3 juvenile males and 2 adult females) received antimicrobial drug therapy (ceftriaxone, 50 mg/kg for adults, 100 mg/kg for infants), 4 by remote delivery and 1 while chemically immobilized. Two untreated animals (1 adult female and 1 male infant born to a symptomatic mother) died. On June 30, the adult female was first observed coughing and lethargic but still feeding. On July 3, she left her night nest in the morning but did not join her group; she exhibited severe clinical signs and was found dead on July 4 at ≈1:00 pm. The infant was 3 days old when it died on July 23. Clinical signs of respiratory illness had not been observed, although its mother showed severe clinical signs for 2–3 days before and after delivery; before delivery, she had received antimicrobial drugs by remote delivery (neither she nor her infant were handled by humans).

Gross postmortem examinations revealed bronchopneumonia in the adult and unilateral pulmonary congestion and an empty stomach in the infant. Formalin-fixed (10%) postmortem tissue samples from the adult and infant were prepared in 6 μm sections for histologic studies, stained with hematoxylin and eosin according to standard methods, and examined by light microscopy. Histologically, the respiratory tract of the adult was characterized by moderate mononuclear tracheitis, laryngitis, and air sacculitis; severe pulmonary alveolar histiocytosis; multifocal severe suppurative pneumonia; and multifocal pulmonary thrombosis and hemorrhage. One section of lung from the infant showed pulmonary atelectasis, congestion, mild alveolar hemorrhage, and histiocytosis. The infant also had moderate neutrophil and macrophage infiltration of the umbilicus at the body wall; neutrophilic inflammation in the media and adventitia of 1 umbilical artery at the level of the bladder; and mild, unilateral, focal, segmental, neutrophilic glomerulitis and tubulointerstitial nephritis. Coronary groove and mesenteric fat were absent.

Multiplex PCR analysis for respiratory pathogens indicated sequences of human metapneumovirus (HMPV) in serum; lung tissue; and throat, nose, anus, and vagina swabs from the adult gorilla, and in lung tissue from the infant (Table). *Streptococcus pneumoniae* was detected in lung tissue and in throat and nose swabs of the adult gorilla but not in the infant. *Klebsiella pneumoniae* was also detected in all specimens from the adult gorilla. Microbial loads were determined by quantitative PCR (Table). The sample with the highest viral load, a throat swab from the adult female (6.2 × 10^5^ genome copies/µL), was pyrosequenced, yielding 607,484 reads comprising 3.8 kb of sequence (27.5% of the genome). Simple pairwise analysis indicated that the strain belonged to lineage B2 of HMPV (*4*). Bayesian analysis revealed close relationship of the gorilla virus to human isolates from South Africa (Figure). Sequence information for the reported HMPV has been deposited with GenBank under accession no. HM197719. Detailed methods are available in the Technical Appendix.

**Table:** Results of microbiologic testing of mountain gorilla tissues, Rwanda*

Sample source and no.	Sample type	MassTag RNA panel results	HMPV viral load, genome copies/µL	MassTag DNA panel results	Bacterial load, genome copies/µL	
					*Streptococcus pneumoniae*	*Klebsiella* *pneumoniae*
Adult						
1	Serum	HMPV	2.7 × 10^3^	*K. pneumoniae*	ND	3.2 × 10^7^
2	Buffy coat	Negative	ND	*K. pneumoniae*	ND	4.4 × 10^8^
3	Kidney	Negative	ND	*K. pneumoniae*	ND	6.2 × 10^8^
4	Lung	HMPV	3.2 × 10^2^	*S. pneumoniae, K. pneumoniae*	1.3 × 10^2^	2.0 × 10^7^
5	Heart	Negative	ND	*K. pneumoniae*	ND	1.4 × 10^7^
6	Spleen	Negative	ND	*K. pneumoniae*	ND	9.1 × 10^5^
7	Liver	Negative	ND	*K. pneumoniae*	ND	3.1 × 10^5^
8	Throat swab	HMPV	6.2 × 10^5^	*S. pneumoniae, K. pneumoniae*	5.5 × 10^4^	1.4 × 10^5^
9	Nasal swab	HMPV	2.3 × 10^5^	*S. pneumoniae, K. pneumoniae*	4.3 × 10^4^	2.4 × 10^6^
10	Vaginal swab	HMPV	2.0 × 10^2^	*K. pneumoniae*	ND	6.0 × 10^5^
11	Anal swab	HMPV	4.0 × 10^2^	*K. pneumoniae*	ND	5.5 × 10^2^
12	Purulent discharge	Negative	ND	*K. pneumoniae*	ND	5.2 × 10^2^
Infant						
18	Spleen	Negative	ND	Negative	ND	ND
19	Lung	HMPV	<5.0 × 10^1^	Negative	ND	ND
20	Liver	Negative	ND	Negative	ND	ND
21	Kidney	Negative	ND	Negative	ND	ND

*HMPV, human metapneumovirus; ND, not detected.

**Figure:**
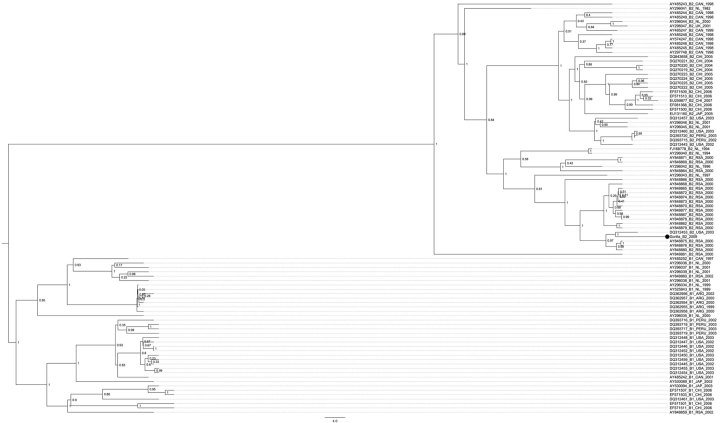
Bayesian analysis of the G gene of human metapneumovirus (HMPV) isolated from an adult female mountain gorilla that died during an outbreak of respiratory disease, Rwanda. Bayesian phylogenetic analyses of sequence differences of the HPMV glycoprotein gene were conducted by using BEAST, BEAUti, and Tracer analysis software packages (http://beast.bio.ed.ac.uk/Main_Page). Only lineage B HMPVs are shown. The black dot indicates the strain newly isolated from the gorilla; trees are rooted at the midpoint; scale is in years.

## Conclusions

Experimental infections of cynomologus macaques with HMPV have suggested that pure infection with this virus causes minimal to mild lesions in conducting airways and increased macrophages in alveoli (*5*). However, paramyxoviruses, including HMPV, can predispose animals to bacterial pneumonia (*6**–**8*), as appeared to be the case in the adult female mountain gorilla reported here. That HMPV can be fatal for gorillas is supported by a report of a respiratory outbreak in wild, human-habituated chimpanzees in which several chimpanzees died (*2**,**9*).

We report conclusive evidence for association of a human virus with death in mountain gorillas (*2**,**3*). Viral RNA in multiple tissue samples from the adult female indicates that she was infected by an HMPV strain at the time of her death. The upper respiratory lesions were suggestive of a viral infection (*9*). The pulmonary lesions indicated a bacterial bronchopneumonia as the proximate cause of death, compatible with an etiologic agent such as *S. pneumoniae* and *K. pneumoniae,* the 2 organisms detected by PCR. Although the cause of death of the infant was likely inanition and acute dissemination of an umbilical infection to a kidney, detection of HMPV as the sole pathogen in the infant tissues supports the presence of this agent in the gorilla group during the respiratory disease outbreak.

The source of the virus is unknown; the strain was most recently described in South Africa. The 2 HMPV-positive animals were not handled by veterinarians or park personnel during the course of their illness. Although HMPV transmission as a result of human intervention to treat sick animals in the group is possible, it does not explain HMPV in the adult female, which died early in the outbreak before any clinical interventions were conducted. Although human proximity to mountain gorillas is essential for their conservation, also crucial is minimizing the risk for human-to–great ape transmission of respiratory pathogens.

## Supplementary Material

Technical Appendix Pathogens included in PCR of samples from mountain gorillas during outbreak of respiratory disease, Hirwa, Rwanda, June 28-August 6, 2009.
