# Supplementary material for: Human Metapneumovirus Infection in Wild Mountain Gorillas, Rwanda
Source: Emerg Infect Dis. 2011 Apr;17(4):711–3. doi: 10.3201/eid1704.100883 (PMC3377396; doi:10.3201/eid1704.100883)
Supplement: Technical Appendix — Pathogens included in PCR of samples from mountain gorillas during outbreak of respiratory disease, Hirwa, Rwanda, June 28-August 6, 2009. [file 10-0883-Techapp-s1.pdf]

Article DOI: 10.3201/eid1704.100883

Human Metapneumovirus Infection in Wild Mountain Gorillas, Rwanda

Technical Appendix

Table. Pathogens included in PCR of samples from mountain gorillas during outbreak of respiratory disease, Hirwa, Rwanda, June 28–August 6, 2009*

Pathogen	Reference
Influenza A virus	(*1*)
Influenza B virus	(*1*)
Respiratory syncytial virus A	(*2*)
Respiratory syncytial virus B	(*2*)
Human coronoavirus OC43	(*3*)
Human coronoavirus 229E	(*3*)
Human parainfluenza virus 1	(*3*)
Human parainfluenza virus 2	(*3*)
Human parainfluenza virus 3	(*3*)
Human parainfluenza virus 4	(*3*)
Human metapneumovirus	(*3*)
Human enterovirus	(*3*)
Human rhinoviruses A, B, and C	(*3*)
Human adenovirus	(*4*)
Chlamydophila pneumoniae	(*5*)
Haemophilus influenzae	(*3*)
Legionella pneumophila	(*3*)
Mycoplasma pneumoniae	(*5*)
Mycobacterium tuberculosis	(*6*)
Neisseria meningitidis	(*6*)
Streptococcus pneumoniae	(*3*)
Acinetobacter baumannii	(*6*)
Candida albicans	(*6*)
Enterobacter spp.	(*6*)
Enterococcus spp.	(*6*)
Klebsiella pneumoniae	(*6*)
Staphylococcus aureus	(*6*)
Methicillin-resistant *S. aureus*	(*6*)
Pseudomonas spp.	(*6*)
Serratia marcescens	(*6*)
Streptococcus pyogenes	(*6*)
Measles virus	This study

*Primer sequences available upon request.

Total RNA was obtained by acid guanidinium thiocyanate-phenol-chloroform extraction (TRI-Reagent; Molecular Research Center, Inc., Cincinnati, OH, USA). New instruments were used for each tissue to prevent cross-contamination. Samples were prepared and analyzed by MassTag PCR for 33 microbes targeting generic influenza A and B viruses, respiratory syncytial viruses A and B, human coronoviruses OC43 and 229E, human parainfluenza viruses 1–4,

human metapneumovirus, human enteroviruses, human rhinoviruses A–C, human adenovirus, *Chlamydophila pneumoniae*, *Haemophilus influenzae*, *Legionella pneumophila*, *Mycoplasma pneumoniae*, *Mycobacterium tuberculosis*, *Neisseria meningitides*, *Streptococcus pneumoniae*, *Acinetobacter baumannii*, *Candida albicans*, *Enterobacter* spp., *Enterococcus* spp., *Klebsiella pneumoniae*, *Staphylococcus aureus*, methicillin-resistant *S. aureus*, *Pseudomonas* spp., *Serratia marcescens*, and *Streptococcus pyogenes* (*3*,*6*). SYBR-Green real time PCRs were used to quantify each of the positive findings. SYBR Green was also used to detect measles virus in singleplex with primers targeting the F gene (Measles_F_FWD, 5′-CGTTGCCACAGCTGCTCA-3′, Measles_F_REV, 5′-TCTCAGATTGTCGATGGCTTGA-3′). Assays were run by using the ABI Prism 7500 Real-time PCR System (Applied Biosystems, Foster City, CA, USA). Programs of the Geneious package (Biomatters, Auckland, New Zealand) were used for sequence assembly and analysis. Sequences were downloaded from GenBank and aligned by using the ClustalX (*7*) implementation in MEGA software (*8*). Bayesian phylogenetic analyses of the sequence differences among the NP, G, and F open reading frames of human metapneumovirus were conducted by using the BEAST, BEAUti, and Tracer analysis software packages (only G is shown) (*9*). Preliminary analyses were run for 10,000,000 generations with the Hasegawa, Kishino, and Yano + gamma distribution nucleotide substitution model to select the clock and demographic models most appropriate for each open reading frame. An analysis of the marginal likelihoods indicated that the relaxed log-normal molecular clock and constant population size model were decisively chosen. Final data analyses included Markov Chain Monte Carlo chain lengths of 30,000,000 generations, with sampling every 1,000 states.

References

1. Schweiger B, Zadow I, Heckler R, Timm H, Pauli G. Application of a fluorogenic PCR assay for typing and subtyping of influenza viruses in respiratory samples. J Clin Microbiol. 2000;38:1552–8. PubMed

2. van Elden LJ, van Loon AM, van der Beek A, Hendriksen KA, Hoepelman AI, van Kraaij MG, et al. Applicability of a real-time quantitative PCR assay for diagnosis of respiratory syncytial virus infection in immunocompromised adults. J Clin Microbiol. 2003;41:4378–81. PubMed DOI: 10.1128/JCM.41.9.4378-4381.2003

3. Briese T, Palacios G, Kokoris M, Jabado O, Liu Z, Renwick N, et al. Diagnostic system for rapid and sensitive differential detection of pathogens. Emerg Infect Dis. 2005;11:310–3. PubMed

4. Avellón A, Pérez P, Aguilar JC, Lejarazu R, Echevarria JE. Rapid and sensitive diagnosis of human adenovirus infections by a generic polymerase chain reaction. J Virol Methods. 2001;92:113–20. PubMed DOI: 10.1016/S0166-0934(00)00269-X

5. Welti M, Jaton K, Altwegg M, Sahli R, Wenger A, Bille J. Development of a multiplex real-time quantitative PCR assay to detect *Chlamydia pneumoniae*, *Legionella pneumophila* and *Mycoplasma pneumoniae* in respiratory tract secretions. Diagn Microbiol Infect Dis. 2003;45:85–95. PubMed DOI: 10.1016/S0732-8893(02)00484-4

6. Palacios G, Hornig M, Cisterna D, Savji N, Bussetti AV, Kapoor V, et al. *Streptococcus pneumoniae* coinfection is correlated with the severity of H1N1 pandemic influenza. PLoS ONE. 2009;4:e8540. PubMed DOI: 10.1371/journal.pone.0008540

7. Thompson JD, Gibson TJ, Higgins DG. Multiple sequence alignment using ClustalW and ClustalX. Curr Protoc Bioinformatics. 2002 Aug;Chapter 2:Unit 2 3.

8. Tamura K, Dudley J, Nei M, Kumar S. MEGA4: Molecular Evolutionary Genetics Analysis (MEGA) software version 4.0. Mol Biol Evol. 2007;24:1596–9. PubMed DOI: 10.1093/molbev/msm092

9. Drummond AJ, Rambaut A. BEAST: Bayesian evolutionary analysis by sampling trees. BMC Evol Biol. 2007;7:214. PubMed DOI: 10.1186/1471-2148-7-214
